# Oxidative Toxicity in Neurodegenerative Diseases: Role of Mitochondrial Dysfunction and Therapeutic Strategies

**DOI:** 10.1155/2011/683728

**Published:** 2011-07-14

**Authors:** Katie Facecchia, Lee-Anne Fochesato, Sidhartha D. Ray, Sidney J. Stohs, Siyaram Pandey

**Affiliations:** ^1^Department of Chemistry & Biochemistry, University of Windsor, 277-1 Essex Hall, 401 Sunset Avenue, Windsor, ON, Canada N9B 3P4; ^2^College of Pharmacy and Toxicology, Long Island University, Brooklyn, NY 11436-1331, USA; ^3^School of Pharmacy and Health Professions, Creighton University Medical Center, Omaha, NE 68178, USA

## Abstract

Besides fluorine, oxygen is the most electronegative element with the highest reduction potential in biological systems. Metabolic pathways in mammalian cells utilize oxygen as the ultimate oxidizing agent to harvest free energy. They are very efficient, but not without risk of generating various oxygen radicals. These cells have good antioxidative defense mechanisms to neutralize these radicals and prevent oxidative stress. However, increased oxidative stress results in oxidative modifications in lipid, protein, and nucleic acids, leading to mitochondrial dysfunction and cell death. Oxidative stress and mitochondrial dysfunction have been implicated in many neurodegenerative disorders including Alzheimer's disease, Parkinson's disease, and stroke-related brain damage. Research has indicated mitochondria play a central role in cell suicide. An increase in oxidative stress causes mitochondrial dysfunction, leading to more production of reactive oxygen species and eventually mitochondrial membrane permeabilization. Once the mitochondria are destabilized, cells are destined to commit suicide. Therefore, antioxidative agents alone are not sufficient to protect neuronal loss in many neurodegenerative diseases. Combinatorial treatment with antioxidative agents could stabilize mitochondria and may be the most suitable strategy to prevent neuronal loss. This review discusses recent work related to oxidative toxicity in the central nervous system and strategies to treat neurodegenerative diseases.

## 1. Sensitivity of Neurons to Oxidative Stress

**Figure 1:**
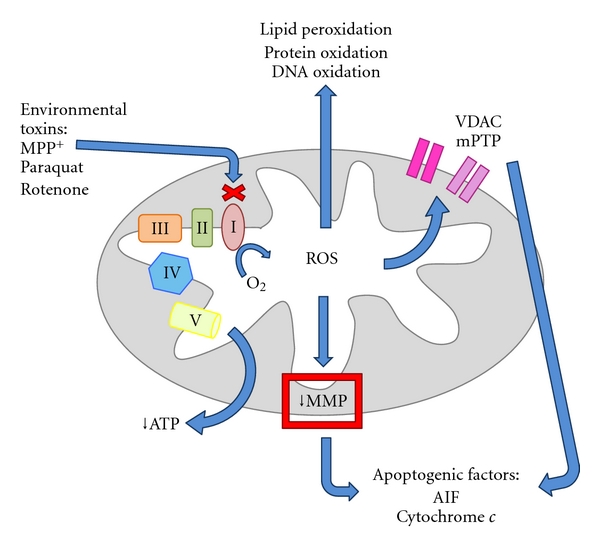
Environmental toxins cause the production of ROS by inhibiting complex I of the electron transport chain (ETC) and decrease the production of ATP. This ROS contributes to a loss in the mitochondrial membrane potential and well as disruption of mitochondrial permeability transition pores and voltage-dependant anion channels contributing to apoptosis. ROS also moves to the cytosol where it oxidizes proteins, DNA, and lipids.

Neuronal cells in the brain are highly sensitive to oxidative stress due to their large dependence on oxidative phosphorylation for energy as compared to other cells. The demand for oxygen consumption is extremely high with 1-2% of the oxygen being converted into superoxide anion radicals (O_2_^∙−^) and hydrogen peroxide, leading to oxidative stress [1]. Oxidative stress exists when there is an imbalance of reactive oxygen species (ROS) production and antioxidant activity. Since there are high levels of metals such as iron in the brain, metal toxicity is also a problem leading to oxidative stress. One way that the brain combats stress is by employing the aerobic isoenzymatic form of lactate dehydrogenase when glucose is metabolized [2]. Previous reports have indicated that these isoenzymatic forms of lactate dehydrogenase play a significant role in the metabolic functions of neurons [3]. Since neurons in the brain also strongly depend on mitochondrial driven aerobic respiration, when the mitochondria become dysfunctional, these neurons become much more susceptible to oxidative stress. Mitochondria already have a high level of oxidative stress and, therefore, any increase in internal or external reactive oxygen species (ROS) leads to dysfunctional mitochondria, which in turn produces more ROS leading to a vicious and detrimental cycle (Figure 1).

Mitochondria have their own enzyme for combating ROS production by converting superoxide radicals to hydrogen peroxide by manganese superoxide dismutase (MnSOD), which are further broken down into water by peroxidases [4]. With heightened levels of oxidative stress, however, these combatants are not enough, and as we age, our defenses against oxidative stress decrease.

### 1.1. Factors Leading to Oxidative Stress

Generation of ROS at complex I, coined “complex I syndrome,” in the mitochondrial electron transport chain (ETC) has been linked to age-associated modifications in the central nervous system [4, 5]. When mitochondrial DNA is the target of oxidation, it can lead to mutations, rearrangements, and transcriptional errors that impair important mitochondrial components leading to more oxidative stress and eventual cell death. This has been shown to be more sensitive in cerebellar granule neuronal cells due to their deficiency in repairing mitochondrial DNA damaged by oxidative stress [4].

Lipid peroxidation causes a collapse of plasma and mitochondrial membranes, releasing cytochrome *c, *and inducing apoptosis. The brain is most affected by lipid peroxidation because of its high oxidizable lipid and metal content in comparison with other tissue [5].

Superoxide radicals and hydrogen peroxide can also create further oxidative stress by metal-catalyzed reactions [6]. Under oxidative stress, superoxide radicals can oxidize iron molecules. The released iron then takes part in the Fenton reaction and generates hydroxyl [6]. It has been shown that inactivation of mitochondrial aconitase (an enzyme involved in the citric acid cycle) by ROS contributes to the release of free iron and hydrogen peroxide leading to neuronal cell [7].

**Figure 2:**
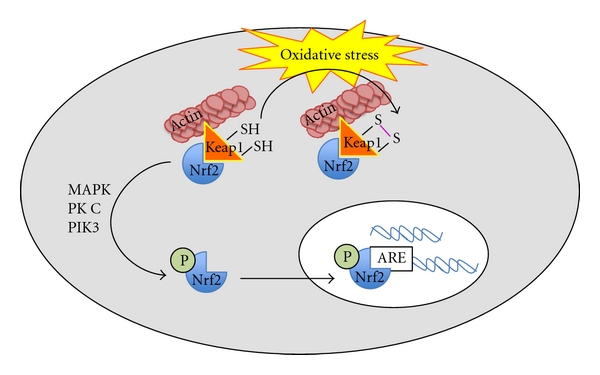
Oxidative stress induces Nrf2 dissociation from Keap1. Nrf2 is activated by phosphorylation and translocated into the nucleus where it may act as a transcription factor for antioxidant response genes.

As a result of the reactions mentioned above, there are increased levels of oxidized glutathione (GSSG) with a concomitant decrease in reduced glutathione (GSH), oxidized protein, and increased lipid peroxidation, all of which are commonly used as markers of oxidative stress and the extent of damage caused by it. We have shown a decrease in GSH levels when rats are challenged with the herbicide paraquat, known to cause neurotoxicity and depletion of substantia nigra neurons due to oxidative stress (Figure 2).

Direct oxidative stress by hydrogen peroxide has been shown to induce inflammation by NF-*κ*B activation and interleukins and is involved in the stress activated protein kinase (JNK) pathway [8]. Recent studies on chronic exposure of neuronal cells to hydrogen peroxide elicit dynamic responses, including changes in cytoskeletal structure, energy metabolic shifts (aerobic to glycolysis), and transmembrane receptor activity [9]. In other studies, chronic exposure to hydrogen peroxide has been shown to have a protective role by inducing the upregulation of antioxidant enzymes such as catalase and superoxide dismutase [10, 11].

### 1.2. Oxidative Stress in Neurodegenerative Diseases Induced by Environmental Toxins

Oxidative stress has been linked to aging and two of the most common neurodegenerative diseases, namely, Alzheimer's disease (AD) and Parkinson's disease (PD). AD is characterized by the loss of neurons, synapses, and neurotransmitters throughout the brain, but especially in the hippocampus and cerebral cortex. Mitochondrial dysfunction may be the underlying reason for the loss, marked by an increase in ROS, lipid peroxidation, and protein oxidation, which are found in AD brains, thus contributing to oxidative stress [28, 29]. Amyloid beta (A*β*), one of the hallmarks of AD, is also involved in oxidative stress and mitochondrial dysfunction by reducing the mitochondrial membrane potential. As an age-related disease, this reduction is intensified in the brain of aging animal models compared to younger animals [28].

In PD, the oxidative stress is most damaging and selective to the mitochondria, specifically in the substantia nigra region of the midbrain. Susceptibility to this disease can be due to genetics, environmental toxins (including most pesticides and herbicides), or a combination of the two, which can cause mitochondrial damage leading to oxidative stress [30].

Many cell culture models have been used to establish the role of oxidative stress in PD in hopes of translating the observed results to an *in vivo* model. For example, glutamate excitotoxicity on mixed neuronal-glial cell cultures along with hypoxia-induced neuronal cell death decreased ATP production and increased ROS [31]. Direct hydrogen peroxide insult has been shown to induce all the same negative factors mentioned above in conjunction with PD [32] and is also associated with the proapoptotic protein Bax [33].

One well-established *in vivo* model for studying PD is the use of 1-methyl-4-phenyl-1,2,3,6-tetrahydropyridine (MPTP), a byproduct found in synthetic heroine. MPTP crosses the blood-brain barrier and is metabolized into 1-methyl-4-phenylpyridinium (MPP^+^) where it has been shown to block complex I of the ETC and increase ROS, lipid peroxidation, and protein oxidation [13, 34]. As mentioned previously, genetics and environmental toxins can provide negative synergistic effects contributing to PD. Recently, it has been found that DJ-1 deficient mice challenged with MPTP have an increase in both oxidative stress and dopaminergic neuronal cell death [35]. DJ-1 is a gene associated with an early onset form of PD.

Exposure to paraquat, which is structurally similar to MPP^+^, has been correlated with the risk of PD in multiple studies [36–38]. Paraquat treatment on human neuronal cell culture causes mitochondrial permeabilization and oxidative stress [39]. It is believed to induce symptoms of PD by reacting in its reduced form with oxygen to produce a superoxide radical [40, 41]. Although banned in Europe in 2007, this herbicide is still the most used worldwide [42, 43]. In addition, rotenone, a pesticide and complex I inhibitor, induces the symptoms of PD similar to paraquat and MPP^+^.

Exposure to paraquat has also been established as an *in vivo* model of PD. Both paraquat-injected rats and mice show parkinsonian symptoms, oxidative stress, and dopaminergic neural loss in the substantia nigra region of the midbrain [13, 44, 45]. These symptoms could also be attenuated by water-soluble coenzyme Q_10_, an ETC component and antioxidant [13]. 

 It was recently shown that the mechanism in which paraquat and rotenone induce dopaminergic cell death might be through the JNK pathway. This is believed to be due to increased phosphorylation of JNK as demonstrated in primary cultured dopaminergic neurons under paraquat and rotenone insult [46, 47] and caspase-3-dependent apoptosis [48]. Another mechanistic pathway of dopaminergic cell death by both paraquat and MPP^+^ is believed to be through the activation of NADPH oxidase-1 (a superoxide-generating enzyme complex), [49, 50]. 

It was also observed that rotenone induced dopaminergic neurodegeneration in an animal model by means of microglial activation, causing NADPH oxidase-derived superoxides to be formed [51]. However, recent studies show that human cell line microglia, although activated, only produce extracellular ROS and, therefore, do not contribute to neurodegeneration when exposed to chronic, low doses of rotenone [52]. Paraquat has been shown to induce oxidative stress in the cytosol, whereas MPP^+^ and rotenone induce oxidative stress in the mitochondria [48]. Although all these chemicals induce symptoms of PD, their mechanism of neuronal cell death varies, therefore, providing multiple approaches to not only study the mechanisms associated with PD but also to develop innovative therapeutic interventions for combating this disease.

### 1.3. Role of Stress-Responsive Transcription Factor Nrf2 (NF-E2-Related Factor 2) in Protection against Oxidative Toxicity

Role of Nrf2, an important stress-responsive transcription factor of the “cap-and-collar” *β*-leucine zipper family, is now considered instrumental to several neurodegenerative disorders [53–55]. Expression of a number of Phase II enzymes (e.g., NQO1, GSTs) and antioxidant proteins (e.g., GCL, HO-1, thioredoxin) are regulated by this gene. It is believed that this process is driven by the association of Nrf2 to the antioxidant responsive element (ARE) consensus sequence (5′-TGACnnnGCA-3′) on the promoter region of these genes [56–59]. Considerable efforts are being made to locate some of its downstream effector genes, including thioredoxin reductase and MafG [60, 61]. However, a clear-cut understanding of the mechanisms of Nrf2 upstream activation remains unclear to date.

It has been shown that Nrf2 is constitutively expressed and localized in the cytosolic compartment and maintains a repressed state by complexing with the actin-associating protein, Keap1. This heterodimerization limits most of Nrf2 to the cytoskeleton and away from the nucleus. Because of a cysteine-rich surface, Keap1 has vulnerability to oxidation during, escalation of intracellular oxidative and nitrosative stress ultimately resulting in global conformational changes to Keap1, thereby, leading to the liberation of Nrf2. After such a reaction, the monomeric Nrf2 becomes available to translocate to the nucleus (Figure 2). In this manner, Keap1 acts as a redox sensor that upregulates ARE antioxidant responses through Nrf2 [16, 56, 62]. Nrf2 activation also has been shown to be mediated through phosphorylation of Nrf2 by mitogen-activated protein kinases (MAPKs), protein kinase C (atypical isoform), and phosphoinositol-3-kinase (PI3K) [54, 63].

In response to oxidative stress, Nrf2 normally translocates from the cytoplasm into the nucleus and transactivates expression of genes with antioxidant activity. Despite this cellular mechanism, severe oxidative damage is not uncommon in Alzheimer (AD) and Parkinson disease (PD). Intense mechanistic investigations in this arena have revealed that Nrf2 expression is abundant in both the nucleus and the cytoplasm of neurons in normal hippocampi with predominant expression in the nucleus. However, in Alzheimer's, Nrf2 was predominantly cytoplasmic rather than nuclear in hippocampal neurons and was not a major component of beta amyloid plaques or neurofibrillary tangles. In contrast, the magnitude of expression of nuclear Nrf2 was much stronger in PD nigral neurons, but it was cytoplasm centric in substantia nigra of normal Alzheimer's. Such observations suggest that Nrf2-mediated transcription is not robust in neurons in AD despite the presence of oxidative stress. But in PD, despite a stronger nuclear localization of Nrf2, the impact of Nrf2 may be inadequate to protect neurodegeneration [53]. Because of this differential Nrf2 expression, it can be considered as a potential therapeutic target for conditions that are sensitive to free radical damage. Unfortunately, these observations do not account for additional contributory roles played by microglia and astrocytes in the overall neuronal system. Future studies may unravel their alleviating or aggravating role during oxidative stress. Nevertheless, mitochondrial dysfunction and buildup of reactive oxygen species are so common in most neurodegenerative disorders that targeting Nrf2 may be a novel way of combating conditions with variable causes and etiologies [64, 65].

## 2. Therapeutic Advances in Alzheimer's Disease and Parkinson's Disease

There are a variety of antioxidants that can attenuate the effects of oxidative stress through multiple mechanisms, and the importance of antioxidants maintaining redox balance is well known. However, no single antioxidant or combination of antioxidants has been discovered to completely halt the progression or cure the diseases that are associated with the destructive properties of oxidative stress. In AD, oxidative stress is a factor throughout the entire brain, making it difficult to find treatment that is specific to the symptoms of the disease. On the other hand, PD is a localized disease, where dopaminergic neurons in the substantia nigra can be monitored, and treatment can be more streamlined and targeted.

Recent studies for the treatment of PD and AD have been directed at agents that target and stabilize the mitochondria [28]. The most promising treatment for AD is the administration of methylene blue, a potential mitochondrial stabilizer at complex I and IV (Table 1). However, to date there is limited amount of published data [66]. 

Antioxidant and anti-inflammatory drugs are the focus of research in combating oxidative stress aimed at stabilizing the mitochondria by quenching ROS generated from the ETC. Common antioxidants such as flavonoids, vitamin C, beta hydroxy acid (BHA), butylated hydroxytoluene (BHT), and nordihydroguaiaretic acid may be unable to access the ubiquinone-binding sites at complex I and II due to their hydrophobicity. These antioxidants are better at combating ROS at the flavin mononucleotide site of complex I [67]. This insight demonstrates that new therapeutic agents need to be specific to the pathophysiological conditions of the site where ROS are generated. Coenzyme Q_10_ has been shown to have some neuroprotective effects and is under clinical trial for Parkinson's disease (Table 1). However, due to its highly hydrophobic nature, the oil-soluble formulation of CoQ_10_ could not be studied in cell culture models and its efficacy in *in vivo* studies is found only at very high doses. A new water-soluble formulation of CoQ_10_, developed by combining it with tocopherol and poly ethylene glycol, has shown very promising results. The water-soluble CoQ_10_ (WS-CoQ_10_) protected human neuronal cells against oxidative stress-induced cell death in several *in vitro* culture models [31, 32, 39]. Furthermore, it has been shown that WS-CoQ_10_ not only decreased the oxidative stress, but stabilized mitochondria and prevented Bax-induced mitochondrial permeabilization [33]. Most importantly, in a recent study with a paraquat-induced Parkinson's disease rat model, WS-CoQ_10_ was shown to be very effective in preventing neuronal loss and amelioration of PD-related symptoms [13]. As shown in Figure 2, levels of oxidative stress induced by paraquat was decreased in WS-CoQ_10_-treated rats.

Recently a disaccharide, Trehalose, has been shown to protect SNpc neurons by the induction of autophagy, short-term reduction of phosphorylated tau and *β*-amyloid plaques in parkin (PK−/−/TauVLW) mouse model [14].

Exercise has been shown to combat oxidative stress in PD by inducing the production of antioxidants and neurotrophic factors [68] and has also been shown to clear A*β* peptides in AD [68, 69].

There are numerous antioxidants on the market that are extremely useful at combating oxidative stress. By assessing and evaluating these antioxidants, it is hoped that they may be used therapeutically for PD, AD, or stroke-related injury.

## 3. Implications of Oxidative Stress in Stroke and Ischemic Related Brain Injury

In ischemic related brain injuries, one of the main perpetrators of cellular damage is oxidative stress. Many studies have indicated that the increase in oxidative stress contributes to lipid damage, protein alterations, and DNA damage. Ironically, the return of blood flow to the infarcted area of the brain causes harm along with its benefits due to the increase in oxygen availability and the increase in oxidative stress that reperfusion causes. In these situations, lactic acid accumulates in the affected neurons promoting prooxidant effects by increasing the H^+^ concentration within the cells and generating more ROS [70]. The primary source of ROS is the superoxide anion radical (O_2_^∙−^), which is generated by leakage from complex III of the electron transport chain of malfunctioning mitochondria [71].

While oxygen may be the main culprit associated with damage due to oxidative stress, it does not act without its accomplices. Hydrogen peroxide is converted to the hydroxyl radical (^•^OH), and the nitric oxide (NO) species that are produced can have extensive implications in neuronal signaling. During the ensuing inflammatory response, O_2_^∙−^ can undergo a lethal reaction with NO to produce the highly detrimental peroxynitrite anion (ONOO^−^), which in turn leads to DNA fragmentation and lipid peroxidation [72]. 

Since the brain makes up only 2% of the total body weight of a human, yet consumes approximately 20% of the available oxygen, it is an excellent environment for the occurrence of oxidative stress [73]. The brain also contains high levels of lipids while possessing low amounts of antioxidants, thus further increasing its susceptibility to damage as the result of ROS and oxidative stress [74].

Stroke is a leading cause of death and long-term disability in industrialized nations [75, 76] and is a condition that regularly leaves its victims in a state of impaired cognition and motor deficits, with nearly 40% of patients not expected to make a full recovery [70]. The damage and detrimental effects of stroke are heavily influenced by oxidative stress and the production of free radicals. 

Two types of stroke can occur, hemorrhagic stroke, and the more common, ischemic stroke. In hemorrhagic stroke, rupture of an artery results in uncontrolled bleeding to the affected area of the brain. In ischemic stroke, there is a blockage of blood flow to the brain due to the formation of a blood clot. This deprivation of oxygenated blood results in the formation of the ischemic core where cells die rather quickly and irreversibly due to necrosis. The onset of lipolysis, protein degradation, and the breakdown of ion homeostasis are some of the events responsible for the rapid death of these cells [77]. 

In the area between the unaffected brain and the ischemic core lies a region where the struggle between the life and death of neurons ensues. This region of the brain is known as the penumbra. It is here that the brain is composed of damaged and malfunctioning, yet salvageable tissue. Cells in this region are susceptible to a programmed form of cell death known as apoptosis. These cells can remain viable and for several days following the onset of stroke [78]. 

Here in the penumbra region is where a host of events related to oxidative stress take place. Ironically enough, reperfusion acts as a double-edged sword. While reperfusion is essential to save the cells affected by ischemia, it also brings along with it its own threat. When reperfusion occurs, there is a large and rapid influx of oxygenated blood to the infarct region. While this delivers the necessary blood, it also brings with it the elements necessary for producing ROS that contribute to the oxidative stress placed upon the already damaged brain tissue. 

When platelets are exposed to conditions of reperfusion, they generate additional ROS in the form of O_2_^∙−^ and ^•^OH. Furthermore, the ROS that are produced during reperfusion are responsible for the activation and transcription of many proteins. For example, ROS stimulate the production of JNK and mitogen-activated protein kinase phosphorylation (p38 MAPK) in the affected neurons of the penumbra. JNK-1 is favored in the nucleus of neurons during reperfusion, and activator protein-1 (AP-1) binding is also enhanced [78]. The activation of AP-1 is necessary for the induction of apoptosis to occur [79]. This action, along with the activation of caspase-3, are several examples of how reperfusion is responsible for initiating cell death within the neurons of the penumbra by controlling the expression of certain genes. 

Along with their role in effecting the transcription of various proteins, ROS generated by reperfusion can itself cause direct cellular stress. Reperfusion causes such a large influx of oxygen that all of it cannot be used by the mitochondria, and normal radical scavenging mechanisms such as superoxide dismutase (SOD), glutathione peroxidase, and catalase are overwhelmed and cannot adequately quench the multitude of free radicals that are produced and leak from the system [80]. The cells of the penumbra are already vulnerable to damage, and the generation of ROS exacerbates the damage that may have already occurred to these cells by lipid peroxidation. In particular, phospholipid membrane degradation is a major concern. The brain is especially susceptible to such damage due to the large amount of lipids that compose its structure. Lipid peroxidation targets the polyunsaturated fatty acids (PUFAs) in the brain, thus, decreasing the membrane integrity. The decrease in membrane stability is especially important because the membrane contains receptor proteins and ion channel entities. Along with its own deleterious effects, lipid peroxidation is also responsible for the inhibition of lipid repair enzymes such as lysophosphatidylcholine acyltransferase and fatty acyl CoA-synthase [81]. 

While cells in the infarct region die instantly via necrosis, cells of the penumbra are likely to die by means of apoptosis. Apoptosis is a programmed form of cell death where the cell expends energy towards its own demise. It is controlled by a complex interconnection of proteins and is often triggered by oxidative stress and the release of cytochrome *c* from the mitochondria [82]. The increased level of ROS is involved in generating the signal that causes permeabilization of the mitochondrial membrane, and, thus, the release of cytochrome *c* into the cytosol. Once this occurs, the initiation of the cascade of caspases occurs. Activation of caspases 3, 8, and 9 will eventually lead to the death of the cell and other surrounding cells [83].

### 3.1. Stroke-Induced Inflammation

Inflammation is a biological response to harmful stimuli and often occurs as a result of stroke. One of the key contributors to the inflammatory response are glial cells, more specifically microglia, that secrete proinflammatory cytokines and chemokines that contribute to the damage done to the penumbra. The most common contributors to the damage due to inflammation are tumour necrosis factor alpha (TNF-alpha) and interlenukin-B (IL-Beta), among others [84]. These cytokines are responsible for the increased expression of cellular adhesion molecules (CAMs) that in combination with platelets, adhere to vessel walls causing a “no-flow” constriction [85] and the release of more proinflammatory molecules. Ultimately, the inflammatory response results in decreased blood brain barrier function, increased cerebral edema, and cell death [84].

### 3.2. The Role of Proapoptotic Proteins in Stroke

As previously mentioned, apoptosis is controlled by a wide range of proteins. Oxidative stress can cause the activation of p53-tumor suppressor gene which in turn is responsible for the increased transcription of Bcl-2 associated X protein (Bax) and the p53 upregulated modulator of apoptosis (PUMA) [86]. PUMA has been shown to be able to interact with the Bcl-2 family of proteins to assist in initiating apoptosis. It has been shown that PUMA is able to associate with the mitochondrial membrane along with Bax to promote the release of cytochrome *c* [87]. Studies involving the PD-associated DJ-1 gene indicate that this gene protects the cells against excitotoxicity and the effects of ischemia. DJ-1 was found to decrease the presence of oxidative stress markers, and, thus, protect the cell due to the alleviation of the effects of oxidative stress [88]. 

 A large majority of the proteins responsible for the balance between death and survival belong to the Bcl-2 family of proteins that protect cells from apoptosis induced by a wide variety of stimuli. One of these proteins is the proapoptotic protein Bax which exists in the cytosol as a harmless 24 kDa monomer. However, in cases of increased oxidative stress, the protein product of the p53 tumor suppressor gene causes increased transcription of Bax to occur [89]. In response to this increased amount of Bax due to oxidative stress, Bax undergoes dimerization either with itself or other members of the Bcl-2 family (e.g., tBID) through interactions of alpha helix 2 with the BH3 domain [90]. This dimerized form of Bax then begins its migration towards the mitochondria. Once in range of the mitochondria, the dimerized form of Bax may associate with the protein transition pore (PTP) of the voltage-dependent anion channel (VDAC) of the mitochondrial membrane. This action allows for the uncontrolled flow of cytotoxic factors, such as cytochrome *c*, to be released from the mitochondria into the cytosol, and inevitably, the demise of the mitochondria [91]. 

Studies have demonstrated that Bax channel activity is necessary for apoptosis to occur since cell death was halted with the use of Bax channel blockers [92]. Since Bax is an essential protein in the regulation of apoptosis, it is an excellent target candidate for therapeutic approaches. Not only does its extensive involvement in the process of cell death make Bax a good therapeutic target, its position in the apoptosis cascade does as well. While focusing on antioxidants may be a valid point of investigation, bolstering of the antioxidant defense machinery still results in permeabilized mitochondria. Blocking the initiation of apoptosis further up the chain by inhibiting Bax function may save the mitochondria and halt apoptosis.

### 3.3. Experimental Models of Stroke

In the cellular model of stroke, an excellent way to mimic ischemic assault is by inducing hypoxia. Hypoxia is the deprivation of an adequate amount of oxygen to tissues, usually accompanied by detrimental effects. To accomplish this in a cellular model, cells can be placed in an oxygen-free chamber for a period of time before being removed [93]. It was found that when conditions of hypoxia exist, hypoxia-inducible factor alpha (HIF-1*α*) expression increases [94] and, therefore, is implicated in hypoxia-induced apoptosis. HIF-1*α* participates by stabilizing the structure of the tumor suppressor gene p53, which leads to the expression of apoptosis-related genes [94, 95]. 

 HIF-1*α* has also been shown to have antiapoptotic effects because those cells with increased levels of HIF-1*α* show resistance to hypoxia-induced apoptosis [96]. The deciding factor of whether HIF-1*α* is protective or harmful to a cell seems to depend on the level of hypoxia. If conditions of mild hypoxia exist, HIF-1*α* is phosphorylated and associated with HIF-1*β*, and the transcription of p53 is low with anti-apoptotic genes being activated [97, 98]. However, in high hypoxic conditions, the reverse is the case. HIF-1*α* is dephosphorylated and p53 levels are unregulated, eventually leading to the activation of pro-apoptotic proteins such as Bax [97, 99, 100]. This is similar to conditions of ischemic stroke where up to 24 hours after ischemia, most pro-apoptotic genes are upregulated, whereas 48 hours to 8 days after ischemia anti-apoptotic genes are the majority of those induced [94]. 

There are a variety of different *in vivo* models of stroke ranging from middle cerebral artery occlusion (MCAO) [101] and four vessel carotid artery occlusion [102]. One model that our laboratory has employed to investigate the effects of stroke in a rat model is the bilateral carotid artery occlusion and two vessel occlusion hypovolemic hypotension (2VO/HT) model [103]. In this model, global forebrain ischemia is induced by occluding the 2 carotid arteries and removing a certain volume of blood from the animal to maintain a mean arterial pressure of 50 mm Hg. This results in an interruption of blood flow to the brain, successfully creating an infarct region similar to stroke and the surrounding penumbra. This model can be used to accurately investigate the effects of various therapeutic reagents and their abilities to protect neurons under conditions similar to stroke.

### 3.4. Therapeutic Approaches for Stroke

At this time, the only known treatment for victims of stroke is the use of thrombolytics, most commonly, tissue plasminogen activator. The downfall to this avenue of treatment is that it must be administered within 3 hours of the onset of stroke. This is relatively hard to accomplish, as most stroke victims do not arrive at hospital to receive treatment within this timeframe. Also, thrombolytics can lead to an increased likelihood of hemorrhages occurring within the brain [85]. Hypothermia has also been investigated as a possible treatment for stroke. It has been found that lowering the body temperature of a stroke victim may improve the neurological outcome [104]. However this technique remains highly experimental as the temperature, duration, and onset of cooling still remains to be accurately determined.

An emerging field of study for treatment of ischemia includes the use of bone marrow stromal cells (BMSC). These cells can differentiate into neural and glial cells, both *in vivo* and *in vitro*, after being transplanted into animal model brains following neurological insult such as intracerebral hemorrhage (ICH) [105]. These neural stem cells migrate to the area of the brain that is injured in order to replace the neuron deficit that was lost due to hemorrhagic stroke. Recent studies have found that these BMSCs are able to generate functional recovery in Wistar rats following ICH [106]. 

Another interesting avenue of exploration into potential therapeutics for stroke is the use of curcumin derivatives. Curcumin has been shown to prevent Alzheimer's markers in animal models of the disease [107] and has also been shown to be effective in reducing the deficits of middle cerebral artery occlusion in a rat [108, 109]. Recent studies have shown that when used as a treatment in a model of stroke, a pyrazole derivative of curcumin was able to enhance memory, contribute to neurotrophic activity, and, most importantly, prevent cell death [110]. 

 Research has focused on blocking pro-apoptotic proteins that are responsible for causing cell death. Recently, advances in treatments for stoke have been made by the use of low molecular weight compounds that inhibit proteins (such as Bax) that are critical in the apoptosis cascade. This is a critical stage for the inhibition of apoptosis due to the fact that Bax channel formation is required for the destabilization of the mitochondria, and subsequent release of cytotoxic factors [92]. These inhibitory compounds were modeled after single-domain antibodies that were able to bind specifically to Bax [93]. They are small enough to have the potential to cross the blood brain barrier and are not susceptible to proteolysis. Recent research completed in our laboratory indicates that these compounds show a high specificity towards the pro-apoptotic protein Bax and are able to block its function and save the neurons of the penumbra from apoptosis [111]. These compounds are able to competitively bind to Bax even when in the presence of single-domain antibodies that are specific to Bax. By binding in some manner to Bax, these compounds prevent the association of Bax with the mitochondria and prevent mitochondrial destabilization, thus, limiting the influx of cytotoxic factors into the cytosol. It is hoped that these compounds will not need-to be administered in such a short-time frame following stroke as is the case for thrombolytics, nor pose the risk of hemorrhage that thrombolytics do. With more investigation, it is likely that the use of low molecular weight compounds will become valid treatment options for stroke patients.

## 4. Conclusion

It is now well established that oxidative stress and mitochondrial dysfunction are the early and key biochemical mechanisms leading to Alzheimer's disease, Parkinson's disease, and stroke-related pathologies. Mitochondria are greatly involved in neuronal cell death due to the vicious cycle of oxidative toxicity, which causes mitochondrial dysfunction that leads to more ROS and the potential collapse of the mitochondrial membrane. Environmental toxins, amyloid-beta toxicity, and ischemia/hyper-perfusion-related toxicity all lead to oxidative toxicity directly or indirectly by mitochondrial destabilization. Significant progress has been made to inhibit neuronal cell death by using anti-oxidants or blockers of pro-apoptotic proteins, but a combinatorial treatment to reduce oxidative stress and stabilize mitochondria to halt neuronal loss needs to be explored.

## Figures and Tables

**Figure 3:**
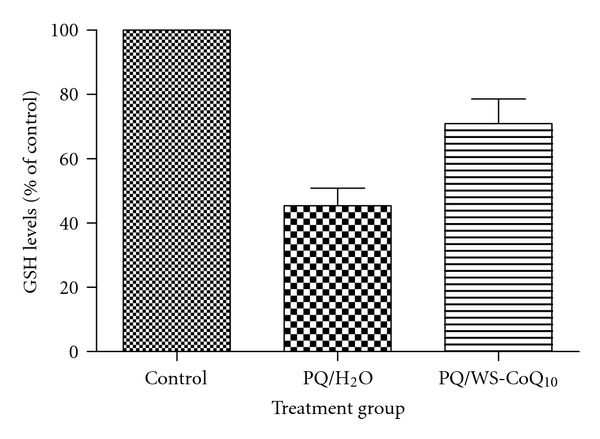
GSH assay. Measuring oxidative stress levels in brain tissue from rats challenged with paraquat and treated with WS-CoQ_10_. GSH levels decrease in the presence of toxin-inducted oxidative stress and are brought back up to control levels in the presence of the antioxidant (WS-CoQ_10_).

**Table 1:** Therapeutic modalities undergoing preclinical/clinical trials for neurodegenerative diseases.

Compound/Chemical	Disease	Effect
CoQ_10_	PD	Reduces the loss of DA neurons in the SNpc (Cleren et al., 2008, [12], Somayajulu-Niţu et al., 2009, [13])

Trehalose	PD	Autophagy-mediated, short-term reduction of phosphorylated tau and *β*-amyloid plaques in parkin (PK−/−/TauVLW) mouse model (Rodríguez-Navarro et al., 2010, [14])

SR-3306 (JNK inhibitor)	PD	Reduces the loss of dopaminergic cell bodies in the SNpc and their terminals in the striatum (Crocker et al., 2011, [15])

Curcumin	PD	Reduces synuclein toxicity, intracellular ROS, and apoptosis in neuroblastoma cells (Dinkova-Kostova et al., 2010, [16])

	AD	Blockes A*β* aggregation (Guo et al., 2010, [17])
		Inhibites A*β* insult (Ono et al., 2004, [18])
		Protects Sprague-Dawley rats from A*β*-induced damage (Frautschy et al., 2001, [19])
		Inhibits neuroglial cell proliferation (Ambegaokar et al., 2003, [20])
		Inhibits A*β*-induced cytochemokine gene expression and CCR5-mediated chemotaxis of THP-1 monocytes by modulating EGR-1 (Giri et al., 2004, [21])
		Inhibits *α*-synuclein aggregation (Pandey et al., 2008, [22])

Methylene blue	AD	Inhibits cGMP pathway
		Attenuates amyloid plaque formation and neurofibrillary tangles (Wischik et al., 2008, [23] Oz et al., 2009, [24])

Viral vector A*β* cDNA (oral vaccination)	AD	Alleviates progressive cognitive impairment with decreased A*β* deposition, insoluble A*β*, soluble A*β* oligomer, microglial attraction, and synaptic degeneration induced in brain regions (Mouri et al., 2007, [25])

AL-108	AD	Stabilizes microtubules and blocks A*β* aggregation (Masters and Beyreuther, 2006, [26])

Curcumin derivative	Stroke	Enhances memory, contributes to neurotrophic activity, and prevents cell death (Lapchak et al., 2011, [27])
